# Discovery of novel 1,3-diaryltriazene sulfonamides as carbonic anhydrase I, II, VII, and IX inhibitors

**DOI:** 10.1080/14756366.2018.1515933

**Published:** 2018-10-09

**Authors:** Suleyman Akocak, Nabih Lolak, Silvia Bua, Claudiu T. Supuran

**Affiliations:** a Department of Pharmaceutical Chemistry, Faculty of Pharmacy, Adiyaman University, Adiyaman, Turkey;; b NEUROFARBA Dept., Sezione di Scienze Farmaceutiche, Università degli Studi di Firenze, Sesto Fiorentino (Florence), Italy

**Keywords:** Carbonic anhydrase, inhibitors, triazene, isozymes, metanilamide

## Abstract

A series of new 1,3-diaryltriazene sulfonamides was synthesised by reaction of diazonium salt of metanilamide (3-aminobenzene sulfonamide) with substituted aromatic amines. The obtained new compounds were assayed as inhibitors of four physiologically and pharmacologically relevant human (h) isoforms of carbonic anhydrases (CA, EC 4.2.1.1), specifically, hCA I, hCA II, and hCA VII (cytosolic isoforms), as well as the tumour-associated membrane-bound isoform hCA IX. All isoforms investigated here were inhibited by the newly synthesised 1,3-diaryltriazene sulfonamide derivatives from the micromolar to the nanomolar range. The cytosolic isoforms were inhibited with K_i_s in the range of 92.3–8371.1 nM (hCA I), 4.3–9194.0 nM (hCA II), and 15.6–9477.8 nM (hCA VII), respectively. For the membrane-bound tumour-associated isoform hCA IX, the K_I-s_ ranged between 50.8 and 9268.5 nM. The structure–activity relationship (SAR) with these newly synthesised metanilamide derivatives are discussed in detail.

## Introduction

Carbonic anhydrases (CAs, also known as carbonate dehydratase, EC 4.2.1.1) are metalloenzymes present in Archaea, prokaryotes and eukaryotes, that catalyse the efficient interconversion of CO_2_ to HCO_3_^−^ and protons via a ping-pong mechanism under physiological conditions^1–6^. This physiologically very simple, but highly relevant reaction plays an important role for the regulation of many physiologic processes in all living organisms. Up to now, seven genetically distinct CA families (α-, β-, γ-, δ-, ζ-, η, and θ-CAs) were described in various taxa, for all of them with numerous isoforms being present in all the investigated organisms^1–6^.

In humans, 15 different isoforms have been described, all belonging to the α-CA family, with some of them being cytosolic (hCA I-III, VII, and XIII), others membrane-bound (hCA IV, IX, XII, and XIV), two mitochondrial (hCA VA and VB), as well as one of them secreted in saliva and milk (hCA VI). Since these isoforms play an important role in acid–base regulation, gluconeogenesis and other biosynthetic reactions, electrolyte secretion, bone resorption/calcification, and tumorigenicity, their inhibition/activation may be exploited in several diseases, including, glaucoma, obesity, neuropathic pain, arthritis, Alzheimers’ disease, and more recently cancer^1–6^.

**Figure 1.**
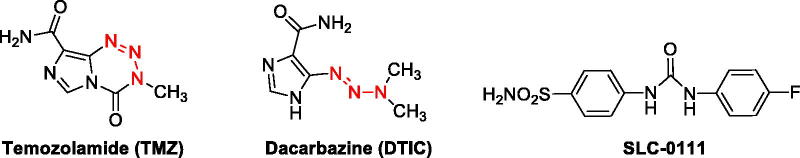
Clinically used triazene substituted compounds (TMZ and DTIC) and efficient CAI SLC-0111 (phase I/II trials for the advanced metastatic solid tumours).

Primary sulfonamides and their isoesters (sulfamides, sulfamates) are the most widely studied CA inhibitors since the late 50’s and some of them have been used as drugs for decades. More recently, one of the sulfonamide-based CA inhibitor (CAI), which is the ureido-substituted derivative SLC-0111 (Figure 1), was possessing a highly effective hCA IX/XII inhibitory action, reached to Phase I/II clinical trials for the treatment of advanced, metastatic solid cancers^7–9^.

Triazenes are a diverse group of compounds which are amenable to many synthetic transformations and are also used for different applications, such as natural product synthesis, combinatorial chemistry, and biomedical applications^10^. On the other hand, triazene compounds of clinical interest (such as Temozolomide and Dacarbazine), are a group of anticancer alkylating agents, with excellent pharmacokinetic properties and limited toxicity^10^ (Figure 1).

**Figure 2.**
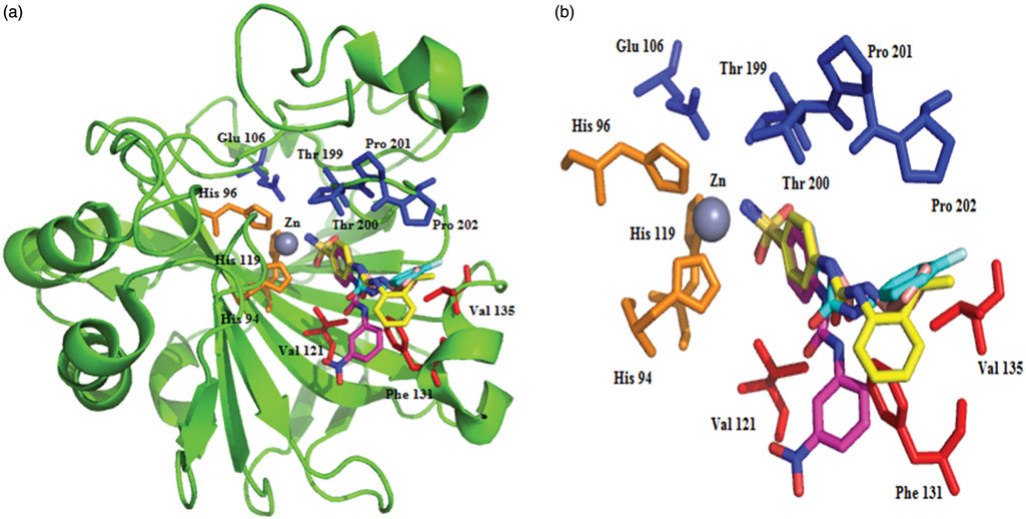
Ribbon diagram (a) and active site detail of the adducts with ureido-substituted benzenesulfonamide CAIs (b); SLC-0111 (cyan, pdb: 3N4B), 4–(3-(3-nitrophenyl)ureido) benzenesulfonamide (pink, pdb: 3N2P), 4–(3-(2-isopropylphenyl)ureido) benzenesulfonamide (yellow, pdb: 3N3J) and 4–(3-cyclopentylureido) benzenesulfonamide (light orange, pdb: 3MZC) (superimposed)^9^. Figure made using PyMol (Delano Scientific).

The X-ray crystal structure of SLC-0111 bound to hCA II as well as of four of its congeners, with various tail moieties was reported^9^. As shown in Figure 2, the benzenesulfonamide fragment of molecules is rather superimposable for the four derivatives, whereas the ureido fragment allows a quite flexible orientation of the tail moieties in various parts of the active site, depending on nature and substitution pattern of the R moiety^9^. This has as a consequence the fact that some of these compounds show a rather impressive isoform specificity. For example, SLC-0111 is an effective inhibitor of only hCA IX, XII, and XIV, being a weak inhibitor of off-target isoforms such as hCA I, II, or IV.^9^

**Figure 3.**
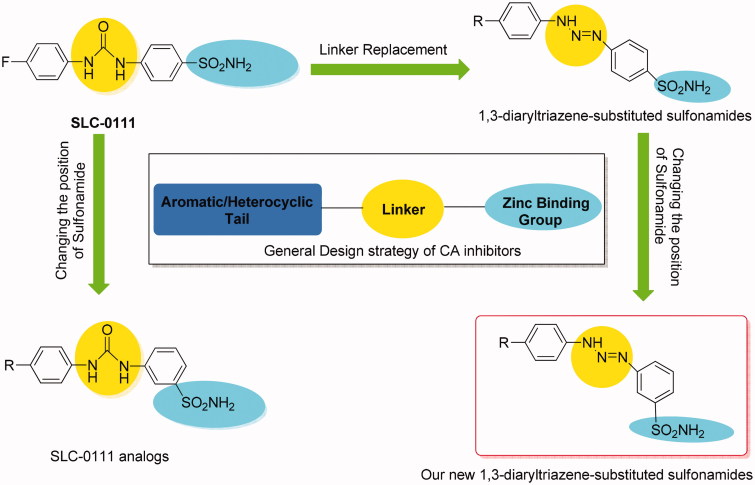
General CA inhibitor design structure and design strategy of the reported 1,3-diaryltriazen-substituted sulfonamide derivatives starting from SLC-0111.

In continuation of our recent interest in CAIs^11^, in this work, we report the synthesis and hCA I, II, VII, and IX inhibitory activity of new 1,3-diaryltriazene sulfonamides **4(a**–**h)** obtained from the reaction of the diazonium salt of metanilamide with different substituted aromatic amines (Figure 3).

## Materials and methods

### General

All chemicals and anhydrous solvents were purchased from Sigma-Aldrich, Merck, Alfa Aesar and TCI and used without further purification. Melting points (mp) were determined with SMP30 melting point apparatus in open capillaries and are uncorrected. FT-IR spectra were recorded by using Perkin Elmer Spectrum 100 FT-IR spectrometer. Nuclear Magnetic Resonance (^1^H-NMR and ^13^C-NMR) spectra of compounds were recorded using a Bruker Advance III 300 MHz spectrometer in DMSO-d_6_ and TMS as an internal standard operating at 300 MHz for ^1^H-NMR and 75 MHz for ^13^C-NMR. Thin layer chromatography (TLC) was carried out on Merck silica gel 60 F_254_ plates.

### General procedure for preparation 1,3-diaryltriazene sulfonamide derivatives 4(a-h)

A solution of metanilamide **1** (5 mmol) in 1.5 ml of conc. hydrochloric acid and 3 ml of water was cooled to 0–5 °C, sodium nitrite (7 mmol) in 3 ml of water was added dropwise to this solution during about 15–20 min under continuous stirring. The mixture was stirred about 20 min at 0–5 °C, and diazonium solution was added to aniline solution (prepared by 5 mmol anilines in 5 ml of MeOH) by adjusting the pH around 6–7 with simultaneous addition of saturated sodium acetate. Then, the reaction mixture was stirred 3 h at 0–5 °C and overnight at room temperature in dark. The obtained colorful mixture was filtered off, washed several times with cold water and the crystallized from ethanol. The final desired products **4(a**–**h)** were dried under vacuum, kept under dark and fully characterised by FT-IR, ^1^H-NMR, ^13^C-NMR, and melting points.

#### 3–(3-(4-fluorophenyl)triaz-1-en-1-yl) benzenesulfonamide (4a).

Yield: 85%; Color: light brown; mp: 140–142 °C; FT-IR (cm^−1^): 3333, 3251 (NH_2_), 1599, 1497 (asymmetric), 1314, 1145 (symmetric) (S=O), 1094; ^1^H-NMR (DMSO-d_6_, 300 MHz, δ ppm): 12.73 (s, 1H, –NH–), 7.87 (s, 1H, Ar-H), 7.75–7.48 (m, 5H, Ar-H), 7.46 (s, 2H, –SO_2_NH_2_), 7.28 (t, 2H, *J* = 2.2, Ar-H): ^13^C-NMR (DMSO-d_6_, 75 MHz, δ ppm): 158.3, 151.5, 145.7, 138.6, 130.6, 124.3, 123.6, 119.8, 116.3, 115.8.

#### 4–(3-(3-sulfamoylphenyl)triaz-2-en-1-yl) benzoic acide (4b).

Yield: 70%; Color: yellow; mp: 161–163 °C; FT-IR (cm^−1^): 3373, 3245 (NH_2_), 1605, 1526, 1405 (asymmetric), 1335, 1161 (symmetric) (S=O), 1097; ^1^H-NMR (DMSO-d_6_, 300 MHz, δ ppm): 12,88 (br.s, 1H, -COOH), 12.75 (s, 1H, –NH–), 7.92 (s, 1H, Ar-H), 7.79–7.52 (m, 5H, Ar-H), 7.46 (s, 2H, –SO_2_NH_2_), 7.30 (t, 2H, *J* = 2.3, Ar-H): ^13^C-NMR (DMSO-d_6_, 75 MHz, δ ppm): 179.6, 159.5, 151.2, 146.2, 139.5, 130.8, 124.7, 123.3, 119.5, 116.2, 115.1.

#### 3–(3-(4-cyanophenyl)triaz-1-en-1-yl) benzenesulfonamide (4c).

Yield: 75%; Color: light yellow; mp: 170–172 °C; FT-IR (cm^−1^): 3369, 3266 (NH_2_), 2218 (CN), 1606, 1521 (asymmetric), 1326, 1139 (symmetric) (S=O), 1094; ^1^H-NMR (DMSO-d_6_, 300 MHz, δ ppm): 13.01 (s, 1H, –NH–), 8.01 (s, 1H, Ar-H), 7.85–7.72 (m, 5H, Ar-H), 7.71–7.61 (m, 2H, Ar-H), 7.50 (s, 2H, –SO_2_NH_2_),: ^13^C-NMR (DMSO-d_6_, 75 MHz, δ ppm): 160.1, 151.8, 146.6, 139.8, 131.0, 125.2, 123.9, 119.8, 118.2, 116.6, 115.3.

#### 3–(3-(4-butoxyphenyl)triaz-1-en-1-yl) benzenesulfonamide (4d).

Yield: 78%; Color: brown; mp: 140–143 °C; FT-IR (cm^−1^): 3362, 3267 (NH_2_), 1596, 1503 (asymmetric), 1333, 1147 (symmetric) (S=O), 1092; ^1^H-NMR (DMSO-d_6_, 300 MHz, δ ppm): 12.92 (s, 1H, –NH–), 7.96 (s, 1H, Ar-H), 7.83–7.70 (m, 5H, Ar-H), 7.69–7.62 (m, 2H, Ar-H), 7.49 (s, 2H, –SO_2_NH_2_), 3.92 (t, 2H, –OCH_2_CH_2_CH_2_CH_3_), 2.00–1.95 (m, 2H, –OCH_2_CH_2_CH_2_CH_3_), 1.68–1.60 (m, 2H, -OCH_2_CH_2_CH_2_CH_3_), 0.98 (t, 3H, –OCH_2_CH_2_CH_2_CH_3_): ^13^C-NMR (DMSO-d_6_, 75 MHz, δ ppm): 159.8, 151.4, 146.1, 139.5, 131.3, 125.7, 123.2, 119.5, 116.2, 115.5, 64.8, 32,5, 19.9, 15.7.

#### 3–(3-(4-methoxyphenyl)triaz-1-en-1-yl) benzenesulfonamide (4e).

Yield: 82%; Color: dark red; mp: 126–128 °C; FT-IR (cm^−1^): 3337, 32578 (NH_2_), 1598, 1498 (asymmetric), 1303, 1147 (symmetric) (S=O), 1092; ^1^H-NMR (DMSO-d_6_, 300 MHz, δ ppm): 13.00 (s, 1H, –NH–), 7.99 (s, 1H, Ar-H), 7.82–7.75 (m, 5H, Ar-H), 7.73–7.66 (m, 2H, Ar-H), 7.51 (s, 2H, –SO_2_NH_2_),: ^13^C-NMR (DMSO-d_6_, 75 MHz, δ ppm): 159.7, 150.8, 146.7, 139.3, 131.2, 125.7, 123.6, 119.5, 116.8, 115.5, 55.3.

#### 3–(3-(3,5-dimethylphenyl)triaz-1-en-1-yl)benzenesulfonamide (4f).

Yield: 78%; Color: orange; mp: 162–164 °C; FT-IR (cm^−1^): 3381, 3264 (NH_2_), 1602, 1488 (asymmetric), 1307, 1137 (symmetric) (S=O), 1090; ^1^H-NMR (DMSO-d_6_, 300 MHz, δ ppm): 8.13 (s, 1H, Ar-H), 7.95–7.66 (m, 2H, Ar-H), 7.72–7.66 (m, 2H, Ar-H), 7.51 (s, 2H, –SO_2_NH_2_), 6.45 (s, 2H, Ar-H), 2.50 (s, 6H, -CH_3_): ^13^C-NMR (DMSO-d_6_, 75 MHz, δ ppm): 160.3, 151.2, 146.9, 139.6, 131.6, 125.8, 123.5, 119.8, 116.2, 115.1, 21.8.

#### 3–(3-(3,4-dimethoxyphenyl)triaz-1-en-1-yl)benzenesulfonamide (4g).

Yield: 75%; Color: dark brown; mp: 113–115 °C; FT-IR (cm^−1^): 3311, 3215 (NH_2_), 1621, 1549, 1489 (asymmetric), 1378, 1150 (symmetric) (S=O), 1089; ^1^H-NMR (DMSO-d_6_, 300 MHz, δ ppm): 8.35 (s, 1H, Ar-H), 8.17–8.12 (m, 1H, Ar-H), 7.76–7.59 (m, 3H, Ar-H), 7.52 (s, 2H, –SO_2_NH_2_), 7.40 (s, 1H, Ar-H), 6.47 (s, 1H, Ar-H), 3.88 (s, 3H, -OCH_3_), 3.80 (s, 3H, -OCH_3_),: ^13^C-NMR (DMSO-d_6_, 75 MHz, δ ppm): 160.8, 151.7, 146.5, 139.3, 138.2, 131.3, 130.2, 125.3, 123.2, 119.3, 116.8, 115.5, 55.8, 55.6.

#### 3–(3-(3,4-dichlorophenyl)triaz-1-en-1-yl)benzenesulfonamide (4h).

Yield: 68%; Color: light yellow; mp: 122–124 °C; FT-IR (cm^−1^): 3415, 3332, 3322 (NH_2_), 1602, 1524, 1470 (asymmetric), 1323, 1150 (symmetric) (S=O), 1121; ^1^H-NMR (DMSO-d_6_, 300 MHz, δ ppm): 8.38 (s, 1H, Ar-H), 8.21–8.16 (m, 1H, Ar-H), 7.75–7.60 (m, 3H, Ar-H), 7.50 (s, 2H, –SO_2_NH_2_), 7.44 (s, 1H, Ar-H), 6.48 (s, 1H, Ar-H): ^13^C-NMR (DMSO-d_6_, 75 MHz, δ ppm): 161.1, 152.5, 146.9, 139.8, 138.5, 131.7, 130.5, 125.6, 123.1, 119.5, 116.3, 115.1.

### CA inhibition assay

An SX.18 MV-R Applied Photophysics (Oxford, UK) stopped-flow instrument has been used to assay the inhibition of various CA isozymes^12^. Phenol Red (at a concentration of 0.2 mM) has been used as an indicator, working at the absorbance maximum of 557 nm, with 10 mM Hepes (pH 7.4) as a buffer, 0.1 M Na_2_SO_4_ or NaClO_4_ (for maintaining constant the ionic strength; these anions are not inhibitory in the used concentration), following the CA-catalyzed CO_2_ hydration reaction for a period of 5–10 s. Saturated CO_2_ solutions in water at 25 °C were used as substrate. Stock solutions of inhibitors were prepared at a concentration of 10 mM (in DMSO-water 1:1, v/v) and dilutions up to 0.01 nM done with the assay buffer mentioned above. At least seven different inhibitor concentrations have been used for measuring the inhibition constant. Inhibitor and enzyme solutions were preincubated together for 10 min at room temperature prior to assay, in order to allow for the formation of the E–I complex. Triplicate experiments were done for each inhibitor concentration, and the values reported throughout the paper is the mean of such results. The inhibition constants were obtained by nonlinear least-squares methods using the Cheng–Prusoff equation, as reported earlier, and represent the mean from at least three different determinations^13–17^. All CA isozymes used here were recombinant proteins obtained as reported earlier by our group^18^^,^^19^.

## Results and discussion

### Chemistry

The rationale behind the design of these new 1,3-diaryltriazene sulfonamide derivatives presented in this work is based on our recent report^11^, in which we showed that novel 1,3-diraryltriazene-substituted sulfonamide derivatives possess interesting CA inhibitory properties. These compounds showed potent inhibition against the cytosolic hCA II, with great selectivity versus hCA I, hCA VII, and hCA IX inhibition. Thus, we decided to apply the same procedure by changing of position of the sulfonamide moiety from *para* to *meta*, in order to investigate whether the potency comes from triazene linker or the position of the sulfonamide zinc-binding group.

**Scheme 1.**
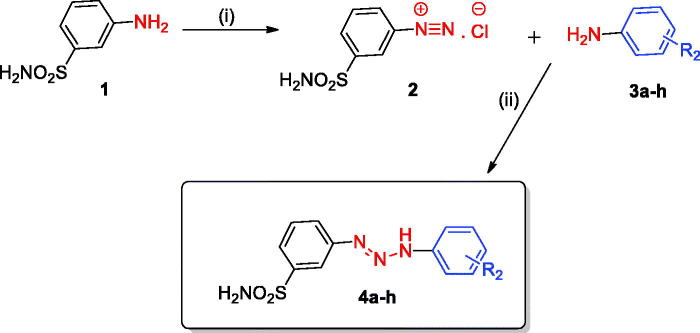
General synthetic route for the synthesis of the 1,3-diaryltriazene sulfonamide derivatives **4(a**–**h)**. Reagent and conditions: (i) H_2_O, HCl, NaNO_2_, 0–5 °C, 30 min, (ii) Substituted aromatic anilines **3(a**–**h)**, MeOH, H_2_O, sodium acetate, 0–5 °C 3h, r.t. overnight.

A series of structurally diverse 1,3-diaryltriazene sulfonamide derivatives were synthesised according to general synthetic route shown in Scheme 1^11^^,^^18^. Briefly, the diazonium salt derived of metanilamide was reacted with different substituted aromatic amines, leading to the formation of 1,3-diaryltriazene sulfonamides. The chemical structures of these novel 1,3-diaryltriazene sulfonamide derivatives reported here were confirmed by using several analytical and spectral data (see experimental part for details).

### CA inhibition studies

The newly synthesised 1,3-diaryltriazene sulfonamides **4(a**–**h)** were evaluated as inhibitors of four physiologically relevant CA isoforms, the cytosolic hCA I, hCA II, and hCA VII, and the transmembrane tumour-associated hCA IX, by a stopped-flow CO_2_ hydrase assay^12^. The clinically used sulfonamide acetazolamide (**AAZ**) was used as a positive control.

The following structure-activity relationship (SAR) may be drawn regarding the inhibition data of Table 1, for the series of 1,3-diaryltriazene substituted sulfonamides **4(a**–**h)**:

**Table 1.** *In vitro* hCA I, hCA II, hCA VII, and hCA IX inhibition data with 1,3diaryltriazene-substituted sulfonamides **4(a**–**h)** investigated here, and standard sulfonamide inhibitor Acetazolamide (**AAZ**) by a stopped flow CO_2_ hydrase assay^12^.

Compound	K_I_^a^ (nM)				
	R	hCA I	hCA II	hCA VII	hCA IX
**4a**	4-F	365 ± 15	858 ± 43	9478 ± 348	8051 ± 126
**4b**	4-COOH	92.3 ± 7.1	68.7 ± 5.1	967 ± 54	835 ± 47
**4c**	4-CN	501 ± 31	605 ± 34	7897 ± 456	8963 ± 348
**4d**	4-BuO	618 ± 40	519 ± 39	5866 ± 314	7428 ± 216
**4e**	4-MeO	416 ± 32	877 ± 62	5761 ± 324	7232 ± 159
**4f**	3,5-diMe	277 ± 18	4.3 ± 0.2	15.6 ± 0.9	50.8 ± 3.6
**4g**	3,4-diMeO	3854 ± 179	6461 ± 310	2557 ± 129	84.9 ± 4.5
**4h**	3,4-diCl	8371 ± 601	9194 ± 504	7834 ± 418	9268 ± 451
**AAZ**	–	250 ± 12	12 ± 0.8	2.5 ± 0.1	25 ± 1.1

a Errors in the range of ± standard error, from three different assays, by a stopped-flow technique.

- The ubiquitous cytosolic isoform hCA I, which is highly abundant among others in the gastrointestinal tract and red blood cells, was moderately inhibited by all compounds investigated here, with inhibition constants in the range of 92.3–8371.1 nM. Compound **4b** (possessing a 4-COOH moiety) showed the best inhibition potency against hCA I, with a K_I_ of 92.3 nM. Interestingly, the 3,4-disubstituted compounds **4g** (3,4-diMeO) and **4h** (3,4-diCl) displayed the lowest hCA I inhibition activity among this series, with K_i_s of 3853.9 and 8371.1 nM, respectively.
- An interesting inhibition profile with the reported 1,3-diaryltriazene-substituted matanilamide derivatives was observed for the physiologically dominant isoform hCA II, for which K_i_s spanning between 4.3 and 9194.0 nM were obtained. The most effective inhibitor was **4f**, which has the 3,5-dimethyl substitution pattern and a K_I_ of 4.3 nM, being almost 3 times more effective compared to the standard inhibitor **AAZ** (Table 1).
- Another cytosolic isoform, hCA VII, mostly present in the brain, was inhibited by most of the new compounds investigated here in the micromolar range, except the compound **4f** which had a K_I_ of 15.6 nM.
- The inhibition potential of novel 1,3-diaryltriazene-substituted metanilamide derivatives **4(a–h)** against hCA IX was not satisfactory since all the compounds reported here were rather inefficient hCA IX inhibitors, with K_I_s in the range of 50.8–9268.5 nM (compared to AZA which has an inhibition constant of 25 nM).

## Conclusions

We investigated a series of 1,3-diaryltriazene-subsituted sulfonamide derivatives as CA inhibitors, continuing our most recent research on 1,3-diaryltriazene based compounds. The compounds were synthesised by reaction of diazonium salt of metanilamide with substituted aromatic amines. The new compounds discovered here were assessed as CAIs, against several pharmacologically relevant isoforms, namely hCA I, hCA II, hCA VII (cytosolic isoforms), as well as membrane-bound tumor-associated isoform hCA IX. Only compound **4f** showed potent inhibition against hCA II and hCA VII with K_i_s of 4.3 and 15.6 nM, respectively. Since hCA II is an important drug target for several diseases such as, glaucoma, retinis pigmentosa, and edema, and hCA VII recently validated antineuropathic pain target, some of these compounds might be improved and used potent CAIs and potential drug candidates.
